# Outcomes in patients with head and neck squamous cell carcinoma with exclusively surgical resection

**DOI:** 10.1016/j.bjorl.2025.101622

**Published:** 2025-05-03

**Authors:** Daniel Naves Araujo Teixeira, Fabio Lau, Vanessa Carvalho de Oliveira, Eduardo Vieira Couto, Thomas Peter Maahs, Carmen Silvia Passos Lima, Carlos Takahiro Chone

**Affiliations:** a Universidade Estadual de Campinas (Unicamp), Departamento de Otorrinolaringologia e Cirurgia de Cabeça e Pescoço, Campinas, SP, Brazil; b Universidade Estadual de Campinas (Unicamp), Departamento de Clínica Médica ‒ Disciplina de Oncologia Clínica, Campinas, SP, Brazil

**Keywords:** Head and neck cancer, Squamous cell carcinoma, Surgery, Survival

## Abstract

**Objectives:**

To estimate patients’ overall and disease-free survival with squamous cell carcinoma of the oral cavity, oropharynx, and larynx, and assess the influence of primary local staging and histopathological features on these outcomes.

**Methods:**

Retrospective data analysis of 102 patients with head and neck squamous cell carcinoma who underwent exclusive surgery as the initial treatment modality. p16 analysis was not evaluated. Exclusion criteria included patients with synchronous or metachronous tumors, those undergoing adjuvant or neoadjuvant chemotherapy or radiotherapy, and those with prior surgery at the exact site. Univariate and multivariate Cox Regression analyses were performed to evaluate outcome-related factors. Survival curves were estimated using the Kaplan-Meier method.

**Results:**

The majority presented early stage I and II (83.4%), T1/T2 tumors (86.2%), N0 (95.1%), and all M0. The primary sites were mainly the larynx (46.1%) and oral cavity (41.2%). The mean overall survival was 71.76 months, with survival rates at 1-, 2-, 3-, 4-, and 5-years of 95.9%, 88.2%, 85.5%, 80.4%, and 74.7%, respectively. The mean disease-free survival was 68.2 months, with rates at 1-, 2-, 3-, 4-, and 5-years of 87.5%, 85.1%, 82.2%, 76.6%, and 69.3%, respectively. Active smoking was associated with a higher risk of death (HR = 9.4, *p* <  0.001) and recurrence (HR = 9.7, *p* <  0.001). Active smoking patients with perineural invasion presented worse overall and disease-free survival. Positive margins were associated with reduced disease-free survival.

**Conclusion:**

Exclusive surgery in the early stages (I and II) emerges as a practical approach. Stage, primary site, margins, perineural invasion, lymphovascular invasion, and smoking cessation were the significant prognostic factors that affected patient outcomes.

**Level of evidence:**

2C.

## Highlights

• The overall survival at 5-years was estimated at 74.7%.
• The disease-free survival at 5-years was estimated at 69.3%.
• Active smoking patients had a 9.4 times higher risk of death.
• Active smoking patients had a 9.7 times higher risk of recurrence.

## Introduction

Cancer is recognized as a public health challenge worldwide due to its significant impact on mortality and reduced life expectancy, encompassing more than a hundred conditions.^1^ Recent statistics reveal that Head and Neck Squamous Cell Carcinoma (HNSCC) is the seventh most common type of cancer globally, with approximately 890,000 new cases annually, representing about 4.5% of all cancer diagnoses worldwide. Additionally, this type of cancer is responsible for approximately 450,000 deaths per year, corresponding to about 4.6% of global cancer deaths.^2^

HNSCC has been increasingly diagnosed in many countries, especially among younger populations, with an annual increase of 30% in incidence projected by 2030. This trend is partly attributed to lifestyle changes such as increased alcohol and tobacco consumption in developing countries, as well as the growing prevalence of oropharyngeal cancer associated with Human Papillomavirus (HPV).^3^

The treatment of HNSCC primarily involves surgery, radiotherapy, and chemotherapy, either alone or in combination, depending on the tumor's location and stage. The goal is to achieve a cure while maintaining functionality. Exclusive surgery can provide oncological control and improve long-term survival rates in patients with small tumors without clinical lymph node involvement or with only a single lymph node, with survival rates ranging from 70% to 90% in patients with early-stage tumors (I or II).^4^ New techniques such as endoscopic endonasal surgery, laser microsurgery, and transoral robotic surgery have expanded indications and increased the likelihood of organ function preservation with excellent oncological control rates.^5^

Thus, in general, early-stage cancers of the oropharynx, hypopharynx, larynx, and sinuses can be treated with exclusive primary surgery, achieving high cure rates and reduced morbidity. Since oral cavity cancers are easily accessible via a transoral approach, surgery is the treatment of choice for these cancers.^6^ This study aimed to estimate overall survival time and disease-free survival time in patients with HNSCC undergoing surgical resection as exclusive therapy and to estimate the influence of primary local staging and anatomopathological characteristics on overall survival time and disease-free survival time in these patients.

## Methods

A retrospective and analytical study was conducted at a tertiary referral center. Through a total of 534 medical records reviewed, 102 patients with HNSCC who underwent surgery as the initial exclusive therapy were selected. These patients were diagnosed in the Otolaryngology and Clinical Oncology outpatient clinics between 2010 and 2019.

Patients eligible for inclusion had squamous cell carcinoma confirmed by histopathological examination in the anatomical locations of the oral cavity, larynx, and oropharynx. They were initially treated with exclusive surgery only. Exclusion criteria included patients with synchronous or metachronous tumors, those who underwent adjuvant or neoadjuvant chemotherapy or radiotherapy, and those with previous surgery in the exact location, metastases from other sites, cutaneous squamous cell carcinomas, and, due to the low number of cases in the sample, nasopharyngeal and hypopharyngeal carcinomas.

Epidemiological data, staging, and patient outcomes were descriptively analyzed. The influence of primary local staging and anatomopathological characteristics on overall and disease-free survival times was also estimated.

The dates of diagnosis, tumor progression or recurrence, death, and last follow-up of the patients included in the study were recorded. Disease-Free Survival (DFS) and Overall Survival (OS) were defined as the interval between the start of treatment and the date of disease progression, death related to the disease, or last follow-up, and between the date of diagnosis and death from any cause or last follow-up, respectively. Additionally, the effects of clinical-pathological aspects on DFS and OS were analyzed.

Exploratory data analysis used summary measures (frequency, percentage, mean, and standard deviation). Groups were compared using the Chi-Square test (categorical variables) or Mann-Whitney test (numerical variables). The Kaplan-Meier estimator was used to estimate overall and event-free survival. Survival of staging groups and primary tumor location were compared using the Log-Rank test. Factors associated with the time to death and recurrence were analyzed using the Cox Regression or Cox Proportional Hazards Model, which models the failure rate among covariates, and the interpretation of the coefficients is given by the Relative Risk (RR or HR). The significance level adopted was 5%.

## Results

Data from 102 patients were obtained, encompassing both sexes and age ranges between 42 and 89 years. The mean age of the patients was 63.2 years with a standard deviation of 10.7 years; 77.5% of the patients were male, 15.7% were active alcohol consumers, 59.8% were former alcohol consumers, 37.3% were active smokers, 57.8% were former smokers, and only 4.9% had never smoked. A personal history of cancer was present in 22.5% of the patients (Table 1).

**Table 1:** Description of the studied patients regarding clinical-demographic and pathological variables.

Variable	*n* (%)
Age	63.2 (10.7)
Gender:	
Female	23 (22.5%)
Male	79 (77.5%)
Alcohol consumption:	
Active	16 (15.7%)
Former drinker (more than 5-years ago)	61 (59.8%)
Never	25 (24.5%)
Smoking:	
Active	38 (37.3%)
Former smoker (more than 5-years ago)	59 (57.8%)
Never	5 (4.90%)
Personal history of cancer:	
No	79 (77.5%)
Yes	23 (22.5%)
Time from clinical presentation to diagnosis (months)	5.74 (3.86)
Time from diagnosis to treatment (months)	2.52 (2.40)
Primary site:	
Larynx	47 (46.1%)
Oral cavity	42 (41.2%)
Oropharynx	13 (12.7%)
Primary site (oral cavity)	
Tongue	16 (38.1%)
Primary site (oropharynx)	
Uvula	4 (30.8%)
Primary site (larynx)	
Glottis	38 (80.9%)
Clinical stage at diagnosis:	
I	52 (51.0%)
II	33 (32.4%)
III	10 (9.80%)
IVA	7 (6.86%)
T at diagnosis:	
T1	54 (52.9%)
T2	34 (33.3%)
T3	9 (8.82%)
T4a	5 (4.90%)
N at diagnosis:	
0	97 (95.1%)
1	2 (1.96%)
2	3 (2.94%)
M at diagnosis: 0	102 (100%)
Degree of tumor differentiation:	
Poorly differentiated	4 (3.96%)
Moderately differentiated	79 (78.2%)
Highly differentiated	18 (17.8%)

Mean (SD) or n (%).

The mean interval between symptom onset and diagnosis was 5.74 months, while the mean interval between diagnosis and the start of treatment was 2.52 months. The more prevalent primary tumor sites were the larynx (46.1%) and oral cavity (41.2%). Most patients presented with stage I or II disease (83.4%), T1 or T2 tumors (86.2%), N0 (95.1%), and all patients were M0. Only 5 (4.9%) patients had clinically positive nodes at diagnosis (Table 1); 32 underwent neck dissection (31.4%) with a mean of 24.6 lymph nodes per patient (Table 2). Of the patients who underwent neck dissection, 10 HNSCC of the larynx, 16 of the oral cavity, and 6 of the oropharynx. Regarding patients who underwent neck dissection with tumor stage T1, 6 had squamous cell carcinoma of the oral cavity, 2 of the oropharynges, and none of the larynx (Table 2). Among the dissected lymph nodes, a mean of 0.81 was positive; 15.7% of patients had positive margins, 58.8% had lymphovascular invasion, 78.4% had perineural invasion, and 19.8% had extracapsular extension. Regarding recurrence, 21.6% of the patients experienced recurrence, 18.6% died, and 84.3% had a complete response (Table 2).

**Table 2:** Description of the studied patients regarding anatomopathological variables and treatment response.

Variable	*n* (%)
Positive margins	
No	86 (84.3%)
Yes	16 (15.7%)
Neck Dissection:	
No	70 (68.6%)
Yes	32 (31.4%)
Neck Dissection:	
Larynx	10 (9.80%)
Oral cavity	16 (15.7%)
Oropharynx	6 (5.88%)
Neck Dissection – T1:	
Larynx	0 (0.00%)
Oral cavity	6 (5.88%)
Oropharynx	2 (1.96%)
Number of dissected lymph nodes	13 (12.7%)
Number of positive lymph nodes	13 (12.7%)
Lymphovascular invasion:	
No	96 (94.1%)
Yes	6 (5.88%)
Perineural invasion:	
No	94 (92.2%)
Yes	8 (7.84%)
Extracapsular spread:	
No	99 (98.0%)
Yes	2 (1.98%)
Recurrence:	
No	80 (78.4%)
Yes	22 (21.6%)
Death:	
No	83 (81.4%)
Yes	19 (18.6%)
Complete response:	
No	16 (15.7%)
Yes	86 (84.3%)

Mean (SD) or n (%).

A comparative analysis of clinical-demographic, pathological, and anatomopathological variables was performed on complete response, death, and tumor stage groups. It was observed that the complete response group had a lower proportion of smokers (*p*-value < 0.001), a lower incidence of stage III or IV, and a lower occurrence of positive margins (*p*-value = 0.007). Active smoking (*p-*value < 0.001) and the presence of perineural invasion (*p*-value = 0.005) were related to death. A higher incidence of patients with positive margins (*p*-value = 0.025), neck dissection (*p*-value < 0.001), and lymphovascular invasion (*p*-value < 0.001) was found in patients with advanced stages (III or IV).

**Fig. 1:**
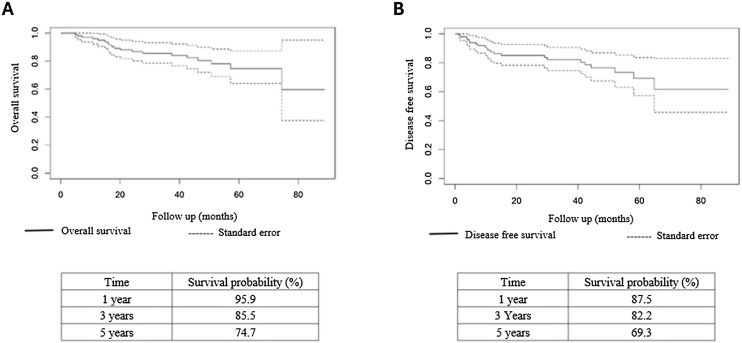
Survival curves ‒ (A) Overall survival time curve; (B) Disease free survival time curve. Source: Original research findings.

**Fig. 2:**
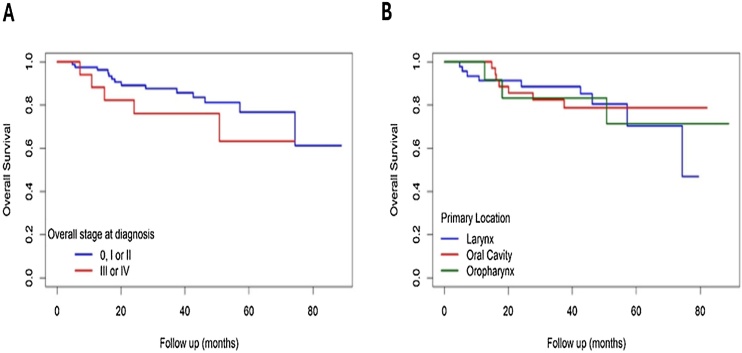
Kaplan-Meier curve off overall survival ‒ (A) Stratified by tumor stage (*p* = 0.300); (B) Stratified by primary tumor site (*p* = 1.000). Source: Original research findings.

The mean overall survival time of the patients was 71.76 months, and disease-free survival at 1-, 3-, and 5-years was estimated at 95.9%, 85.5% and 74.7%, respectively (Fig. 1 A and B). Staging (*p*-value = 0.300, Fig. 2) and primary tumor location (*p*-value = 1.000, Fig. 2) did not significantly affect the overall survival of the patients.

**Fig. 3:**
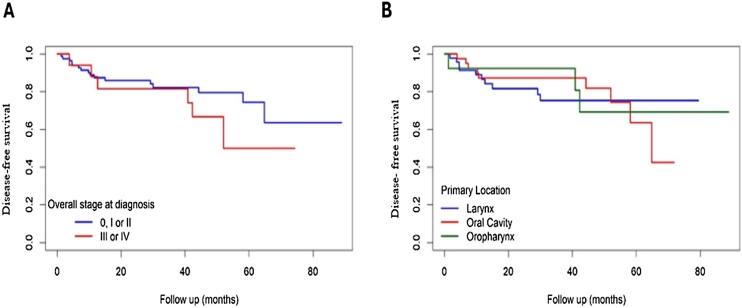
Kaplan-Meier curve of Disease-free survival ‒ (A) Stratified by tumor stage (*p* = 0.300); (B) Stratified by promary tumor site (*p* = 1.000). Source: Original research findings.

The mean disease-free survival time was 68.2 months. Recurrence-free survival was estimated at 87.5%, 82.2% and 69.3% at 1-, 3-, and 5-years, respectively (Fig. 1 A and B). Analyses did not reveal significant changes in event-free survival to staging (*p*-value = 0.300, Fig. 3) and primary tumor location (*p*-value = 1.000, Fig. 3).

The Cox regression was used to assess the influence of clinical-demographic and pathological variables on the risk of death (Table 3). This model showed that active smoking patients had a 9.4 times higher risk of death than patients who quit smoking more than five years (95% CI: 2.7–32.7%; *p*-value < 0.001). Never having consumed ethanol decreased the risk of death by approximately 55%. On the other hand, the presence of a family history of cancer and tumor stages T3 and T4 increased the risk of death by 51% and 75%, respectively. Evaluating all clinical-demographic and pathological variables in a multivariate Cox regression with stepwise variable selection, it was observed that only smoking remained in the model, indicating that among the variables, it is the most important in explaining the risk of death.

**Table 3:** Assessment of factors associated with overall survival (Cox regression).

Variable	Univariate		Multivariate	
	HR (95% CI)	*p*-value	HR (95% CI)	*p*-value
Age	0.96 (0.91–1.00)	0.071		
Time from clinical presentation to diagnosis	0.86 (0.70–1.06)	0.148		
Time from diagnosis to treatment	1.08 (0.93–1.25)	0.340		
Gender Male	0.81 (0.29–2.26)	0.693		
Skin Colour White	0.49 (0.17–1.36)	0.168		
Alcohol Consumption Former (more than 5 years)	0.96 (0.27–3.39)	0.944		
Alcohol Consumption Never	0.54 (0.11–2.7)	0.456		
Smoking Status Active	9.44 (2.73–32.66)	<0.001	9.44 (2.73–32.66)	<0.001
Personal History Yes	0.80 (0.26–2.42)	0.687		
Familial History Yes	1.51 (0.59–3.82)	0.389		
Primary Location Oral Cavity	0.92 (0.34–2.48)	0.869		
Primary Location Oropharynx	1.01 (0.27–3.75)	0.990		
T stage at diagnosis T3 or T4	1.75 (0.62–4.92)	0.288		

It was also shown that active smoking patients had a 9.7 times higher risk of recurrence than patients who quit smoking more than five years (95% CI: 3.2–29.1; *p*-value < 0.001, Table 4). Never having consumed ethanol decreased the risk of recurrence by approximately 23%. In contrast, active ethanol consumption and a family history of cancer increased the risk of recurrence by 24% and 61%, respectively. Evaluating all clinical-demographic and pathological variables in a multivariate Cox proportional hazards model with stepwise variable selection, it was observed that only smoking remained in the model, indicating that among the studied variables, it is the most important in explaining the risk of recurrence.

**Table 4:** Assessment of factors associated with disease-free survival (Cox proportional hazards model).

Variable	Univariate		Multivariate	
	HR (95% CI)	*p*-value	HR (95% CI)	*p*-value
Age	0.95 (0.91‒0.99)	0.026		
Time from clinical presentation to diagnosis	0.91 (0.78–1.06)	0.229		
Time from diagnosis to treatment	1.06 (0.91–1.22)	0.467		
Gender Male	1.05 (0.39–2.86)	0.920		
Skin Colour White	0.77 (0.26–2.28)	0.635		
Alcohol Consumption Former (more than 5 years)	1.24 (0.36–4.31)	0.730		
Alcohol Consumption Never	0.77 (0.17–3.43)	0.728		
Smoking Status Active	9.74 (3.26–29.08)	<0.001	9.74 (3.26–29.08)	<0.001
Personal History Yes	0.66 (0.22–1.98)	0.459		
Familial History Yes	1.61 (0.67–3.91)	0.291		
Primary Location Oral Cavity	0.96 (0.39–2.37)	0.929		
Primary Location Oropharynx	0.87 (0.24–3.2)	0.833		
T stage at diagnosis T3 or T4	0.95 (0.91‒0.99)	0.026		

Overall and disease-free survival is generally higher in the early stages (I or II) than in the advanced stages (III or IV). Both overall and disease-free survival decreased over the years, with lower rates in advanced stages.

The low number of patients who underwent surgery as exclusive therapy is explained by the diagnosis of HNSCC, which is generally detected at more advanced stages, requiring combined therapy. The low number is also justified by the complexity of the service, which receives patients referred from other centers, usually at advanced stages, making a single therapeutic approach unfeasible.

## Discussion

Smoking is a well-established risk factor for HNSCC, with studies confirming a dose-response relationship between smoking habits and the risk of squamous cell carcinoma. A meta-analysis by Koyanagi et al.,^7^ comprising 12 studies, revealed that smokers have a relative risk of 2.43 compared to non-smokers, with a higher risk for active smokers (2.68) and a lower risk for ex-smokers (1.49). Smoking cessation, especially after cancer diagnosis, has shown significant benefits. In the study by Chen et al.,^8^ 41 patients (65%) managed to quit smoking during chemotherapy, which resulted in a significantly higher progression-free survival (*p* =  0.03) compared to patients who continued smoking. The meta-analysis by Caini et al.^9^ also indicated that patients who stopped smoking at or near the time of diagnosis had better overall survival (HR = 0.80, 95% CI 0.70‒0.91). This reinforces the importance of smoking cessation as part of the multidisciplinary treatment for Head and Neck Cancer (HNC).

Perineural invasion is another significant adverse prognostic factor for patients with HNSCC. Studies, including that of Zhu et al.,^10^ showed that the presence of perineural invasion negatively affects overall survival, disease-free survival, and disease-specific survival, with a higher recurrence rate and worse clinical outcomes. The study based on The Cancer Genome Atlas (TCGA)^11^ revealed that 45% of HNSCC patients had perineural invasion, highlighting its prognostic relevance.

According to the Royal College of Pathologists, factors influencing clinical outcomes include the degree of differentiation, the pattern of invasion, the proximity of carcinoma to resection margins, and the presence of extranodal spread. Additionally, a meta-analysis showed that extranodal spread halved the chances of five-year survival (OR = 2.7, 95% CI 2.1–3.7).^12^

In comparison, the results of the present study indicated that 19.8% of patients had extranodal spread, which aligns with the literature pointing to this factor as an important adverse prognostic indicator. The presence of extranodal spread was associated with a significant reduction in survival, highlighting the relevance of this factor in treatment planning and follow-up for patients with squamous cell carcinoma.

Another important factor for prognosis is the status of resection margins. The presence of positive margins was observed in 15.7% of patients in this study, which aligns with the literature that indicates positive margins as a sign of worse prognosis. The study by Haque et al.,^13^ which included 261 cases, confirmed that tumors larger than 2 cm and positive margins are associated with higher mortality. Furthermore, compromised margins or those smaller than 4 mm are strongly associated with reduced disease-free survival and increased local recurrence rates. The findings of this study also support the need for wide margins in patients at high risk for local recurrence, as indicated by current guidelines.

Neck dissection, especially in patients with occult neck lymphadenopathy, continues to be a recommended practice. In this study, 31.4% of patients underwent neck dissection, following recommendations for patients with high risk of occult metastases in the lymph nodes. The prospective study by D’Cruz et al.^14^ which focused on oral cancer, highlighted that elective lymph node dissection, compared to therapeutic dissection, showed a significantly higher overall survival rate (80.0% vs. 67.5%), reinforcing its importance in the treatment of HNSCC patients.

Although this study did not include data on HPV infection, recent literature has highlighted the importance of HPV as a prognostic factor, especially in oropharyngeal cancer. According to Rietbergen et al.,^15^ a high percentage of oropharyngeal squamous cell carcinoma cases are HPV-positive, with an increasing prevalence over the years. HPV infection is associated with better prognostic outcomes, including lower recurrence rates and higher survival.

Regarding survival, the five-year overall survival in this study was 74.7%, and the disease-free survival was 69.3%. These values align with other studies in the literature. For example, the study by Duvvuri et al.,^16^ which reported a response rate of 88.2% for patients in early stages (I and II), is consistent with the findings of this study. The survival rate was significantly lower in advanced stages (III and IV), with a response rate of only 64.7%, emphasizing the importance of early diagnosis to improve prognosis.

This study is limited by its retrospective nature and the low number of patients undergoing exclusive surgery, reflecting the often-late diagnosis of HNSCC. However, the clinical and demographic data presented provide valuable insights into adverse prognostic factors, such as smoking, perineural invasion, resection margins, and the importance of neck dissection in the management of HNC.

Smoking cessation should be considered an integral part of HNC treatment, with benefits extending beyond oncological treatment, offering advantages for the overall health of patients. Public policies that encourage smoking cessation and provide adequate resources are essential to improving patient outcomes. Furthermore, early diagnostic strategies and effective multidisciplinary management are crucial for rehabilitation, with lower morbidity rates and better prognosis for patients with HNSCC.^17^

## Conclusion

Exclusive surgery, when administered in early stages (I and II), emerges as an effective treatment, providing 5-year survival rates. Diagnosis in early stage is important, evidenced by the high rate of complete response optimizing survival. The importance of prognostic factors, such as TNM stage, tumor location, perineural and angiolymphatic invasion, tumor margins, and smoking, is highlighted in patient outcomes.

## CRediT authorship contribution statement

Fabio Lau: Manuscript writing contribution.

Vanessa Carvalho de Oliveira: Manuscript writing contribution.

Eduardo Vieira Couto: Manuscript writing contribution and critical revision of important intellectual content.

Thomas Peter Maahs: Manuscript writing contribution.

Carmen Silvia Passos Lima: Contribution with conception and design, data acquisition, analysis, and interpretation of data.

Carlos Takahiro Chone: Contribution with conception and design, data acquisition, analysis, and critical revision of intellectual content

## Funding

None to declare.

## Declaration of competing interest

The authors declare no conflicts of interest.
